# Prevalence of Obesity in Patients With Type 2 Diabetes Mellitus in Yemen

**DOI:** 10.5812/ijem.13633

**Published:** 2014-04-01

**Authors:** Butheinah A. Al-Sharafi, Abdallah A. Gunaid

**Affiliations:** 1 Department of Medicine, Sana’a University Medical School, Sana’a, Yemen

**Keywords:** Obesity, Overweight, Type 2 Diabetes Mellitus, Body Mass Index, HbA1c

## Abstract

**Background:**

Obesity is common in type 2 diabetic patients in some of the Middle Eastern countries, which are amongst the countries with highest rates of diabetes mellitus and obesity.

**Objectives:**

We conducted this study to assess the prevalence of obesity in Yemeni patients with type 2 diabetes mellitus.

**Patients and Methods:**

Body mass index (BMI) of patients with type 2 diabetes mellitus who were 25-years-old or older was measured during their first visit to an endocrinology and diabetes clinic in Sana’a, Yemen over a 4-year period from May 2007 to May 2011.

**Results:**

The BMI was measured in 1640 patients (721 males and 919 females) who attended the clinic. According to the measured BMI, 328 (45.5%), 314 (43.5%), 79 (11%) of the male patients were non-obese (BMI < 25 kg/m^2^), overweight (BMI 25-29.9 kg/m^2^), and obese (BMI ≥ 30 kg/m^2^), respectively. On the other hand, 256 (28%), 369 (40.0%), and 294 (32%) of the female patients were non-obese (BMI < 25 kg/m^2^), overweight (BMI 25-29.9 kg/m^2^), and obese (BMI ≥ 30 kg/m^2^), respectively.

**Conclusions:**

The prevalence of obesity in patients with type 2 diabetes mellitus in Yemen is high with respect to the Yemeni population, especially in females.

## 1. Background

Obesity is prevalent in patients with type 2 diabetes mellitus (type 2 DM). In some areas such as the United Kingdom, approximately 86% of patients with type 2 DM are overweight or obese (1). In Australia 53% of patients with type 2 DM are obese and 32.8% are overweight (2). In neighboring countries like the Saudi Arabia, the prevalence of the BMI ≥ 25 among patients with type 2 DM is around 87.5% and females had a higher prevalence of the BMI ≥ 25 (87.7%) in contrast to males (83.1%) (3). In Yemen with an approximate population of 23 million, the prevalence of DM is estimated to be 9.75% in individuals aged 20-years-old and older (4). This rate is lower than the prevalence in neighboring Arab countries such as Saudi Arabia (23.1% among patients aged 7 to 80 years) (5), Bahrain (25.7% among patients aged 20 years or older) (6), and Oman (16.1% among patients aged 30 to 64 years) (7).

## 2. Objectives

The aim of this study was to assess the prevalence of overweight and obesity among Yemeni patients with type 2 DM aged ≥ 25 years on their first visit to the endocrine and diabetes clinic. In addition, we aimed to reveal its association with age of the patients, and the duration of the disease.

## 3. Patients and Methods

The Yemeni patients with type 2 DM who attended a specialized diabetes and endocrinology clinic since May 2007 through May 2011 were recruited in the study. Patients of non-Yemeni origin were excluded from the study. BMI (the weight in kilogram divided by height in square meter) was calculated for all patients at the first visit. All the participants had ≥ 25 years of age. According to the BMI, the patients were allocated into 5 groups (BMI < 19, 19-24.9, 25-29.9, 30-34.9, and BMI ≥ 35). The analysis was performed on all patients admitted to the study. Continuous variables were summarized by descriptive statistics, using means and standard deviation (SD), and the difference was measured by t test. Confidence intervals (95% CI) were calculated in order to indicate precision of the sample estimation, variability of the characteristics, and the degree of confidence being studied. Median values and interquartile range (IQR 25-75%) were also used to show the central tendency; its upper and lower quartiles as a preferred measure of range were used with skewed distribution. Categorical variables were expressed as numbers and percentages and the difference was examined by the chi-square test. Pearson correlation coefficient and its P value were used to assess the correlation between variables. All statistical analyses were two-sided, using a pre-specified 5% significance level, i.e. significance was defined as P value < 0.05. Analysis was performed using SPSS software, version 18.0 for windows (SPSS Inc, Chicago, Illinois, USA).

## 4. Results

A total of 1640 Yemeni patients with 25 years of age or older attended to the clinic over a four-year period. All of them were recruited in the study. The median (IQR) duration of DM was 3.0 (0.1-8.0) years and the mean duration was 5.0 ± 6.0 years (95% CI: 4.65-5.27). The mean age of the study population was 50.3 ± 11.5 years, (96% CI: 49.7-50.9). 

Most of the patients presented with recently diagnosed DM. the duration of the disease since the time of diagnosis was less than five years, five to ten years, ten to 15 years, and more than 15 years in 980 (59.7%), 312 (19%), 196 (12%), and 152 (9.3%) patients, respectively. Among the patients, 959 (58.5%) had a BMI ≥ 25 kg/m^2^ and 276 (28.8%) patients were obese (BMI ≥ 30 kg/m^2^).

The basic characteristics of the study population is shown in Table 1. It illustrates that both male and female patients were age-matched and had similar duration of the disease. Although males had significantly higher height and weight than females (P < 0.0001), females were significantly heavier than males with a mean BMI of 28 kg/m^2^ and 25.4 kg/m^2^, respectively (P < 0.001). 

**Table 1.** Basic Characteristics of Study Population

Variables	Male, (n = 721)	Female (n = 919)	P Value
**Age, y**			NS
mean ± SD	50.3 (12.5)	50.7 (10.7)	
95% CI ^a^ of mean	49.4-51.2	50-51.3	
Median (IQR ^a^)	50 (41-60)	50 (45-60)	
**Weight, kg**			< 0.001
mean ± SD	69.2 (12.4)	64.7 (12.9)	
95% CI of mean	68.3-70.1	63.9-65.6	
Median (IQR)	68.9 (60-76.5)	63 (56.3-72)	
**Height, cm**			< 0.001
mean ± SD	164.8 (6.8)	152 (6.7)	
95% CI of mean	164.3-165.3	151.5-152.3	
Median (IQR)	165 (160-169)	152 (148-156)	
**BMI, kg/m^2 ^**			< 0.001
mean ± SD	25.4 (3.9)	28 (5.3)	
95% CI of mean	25.1-25.7	27.7-28.4	
Median (IQR)	25.3 (22.7-27.9)	27.6 (24.4-31)	
**Duration of diabetes**			NS
mean ± SD	4.8 (6.2)	5.3 (6.1)	
95% CI of Mean	4.3-5.2	4.9-5.7	
Median (IQR)	2.0 (0.0-7.0)	3.0 (0.3-8.0)	

^a^ Abbreviation: CI, Confidence interval; IQR, interquartile range.

Table 2 shows sex-specific and age-specific mean BMI values of the study population. Overall, the mean BMI was significantly higher in females (28 ± 5.3 kg/m^2^) than in males (25.4 ± 3.9 kg/m^2^) (P < 0.001).

**Table 2.** Mean BMI Values (kg/m^2^) by Age and Sex for 1640 Yemeni Adults With Type 2 Diabetes Mellitus

Age range, y	Male			Female		
	No.	BMI ^a^, Mean ± SD	SE ^a^	No.	BMI ^a^, Mean ± SD	SE
**Overall (total sample)**	721	25.4 ±3.9	0.14	919	28 ±5.3	0.18
**25 – 34**	78	25.9 ± 3.9	0.44	63	27.6 ± 6.0	0.8
**35 - 44**	144	26.1 ± 4.4	0.37	154	28.9 ± 5.9	0.47
**45 – 54**	217	25.4 ± 3.5	0.24	326	28.4 ± 5.3	0.29
**55 – 64**	174	25.4 ± 3.6	0.27	285	27.6 ± 4.6	0.27
**65+**	108	24.2 ± 4.0	0.38	91	26.8 ± 5.7	0.6

^a^ Abbreviations: BMI, body mass index; SE, standard error

There was a significant difference between the mean BMI for females and males in all age groups. The peak differnce in the mean BMI between females and males was in those aged 45-54. There were significant sex differences with regard to the BMI distributions in patients with type 2 DM. In patients with a low or normal BMI, we found a male preponderance. Among the group with a BMI < 19, there were 29 (4.0%) males out of 721 and 16 (1.7%) out of 919 were females (Yates corrected X^2 ^= 7.0, P = 0.008). Among those with normal weight (BMI, 19-24.9 kg/m^2^), there were 299 (41.5%) out of 721 males and 240 (26.1%) out of 919 females (Yates corrected X^2 ^= 42.5, P < 0.0001). In the overweight group (BMI of 25-29.9 kg/m^2^), 314 (43.6%) out of 721 were males and 369 (40.2%) out of 919 were females (Yates corrected X^2 ^= 1.8, P = 0.18). In the group with mild obesity (BMI of 30-34.9 kg/m^2^), there were 205 (22.3%) out of 919 females 71 (9.8%) out of 721 males (Yates corrected X^2 ^= 43.9, P < 0.0001). In the group with moderate obesity (BMI ≥ 35), we also found a much higher rate of obesity among the females than males with the frequency of 89 (9.7%) out of 919 and 8 (1.1%) out of 721 patients, respectively (Yates corrected X^2 ^= 51.9, P < 0.0001).

In order to have further insight into the association among different variables, we computed Pearson correlation coefficient (r) for exploring the association between BMI and each patient’s age and duration of DM. We also tested the association between duration of DM and glycemic control expressed as HbA1c levels. There was a significant inverse correlation between BMI and age (r = -0.100, 2-tailed P = 0.01), between BMI and duration of DM (r = -0.067, 2-tailed P = 0.01) These results declared that the BMI values tended to be significantly lower with advanced age and increased duration of diabetes.

## 5. Discussion

In this study, the prevalence of BMI ≥ 25 kg/m^2^ in patients with type 2 DM was approximately 58.5% and 28.8% of them were obese (BMI ≥ 30 kg/m^2^). The sex-specific and age-specific mean BMI values were significantly higher in females than in males across different age groups with the peak difference between 45 to 54 years of age. Most of our patients in our study were recently diagnosed. 

With the increasing prevalence of obesity, DM has become a growing problem worldwide. Both DM and obesity are multifactorial diseases of considerable heterogeneity (8). The Eastern Mediterranean region had one of the highest prevalence of obesity worldwide (9). This is particularly true for countries in the region, like Saudi Arabia, where obesity prevalence is 23% in males and 36.4% in females and Egypt, where obesity prevalence is 22% in males and 48% in females (10). On contrary, in less wealthy countries like Sudan and Yemen, the prevalence of obesity is much lower with 1.5% in males and 6.5% in females in Sudan (ages 25-64) (10) and 2.5% in males and 12.4% in females in Yemen aged 20 and older (11). moreover, five Persian Gulf countries are among the countries with the highest prevalence of DM in the world including neighboring countries to Yemen as Saudi Arabia and Oman (among those aged 20-79) (12). In our study, patients with DM who were overweight or obese accounted for more than 64% of the total investigated population with DM. This figure was higher than the findings of a previous study in Yemen that overweight and obesity accounted only for 26.2% of patients with type 2 DM aged 20-65 (13). The worldwide data Analysis concerning the association between BMI and both morbidity and mortality suggested that the association of BMI with most diseases was rather continuous (14) and generally, women had a higher mean BMI than men (15). Therefore, using the mean BMI changed the usual categorical analysis based on the rates of overweight and obesity. Our data indicated that at all ages, females had higher mean BMI than males, and that the overall mean BMI was significantly higher in females than in males (28 vs. 25.4). These results were also higher than the previously published mean BMI of participants from an urban community in Yemen (23.9 and. 21.8 in females and males, respectively) (16). In our study, the prevalence rate of obesity (BMI ≥ 30 kg/m^2^) in females was three times higher than males with type 2 DM (32% vs. 11%). This female-male difference was even higher than the rate reported from Saudi Arabia (87.7% vs. 83.1%) (3). Despite the majority of women denied any special effort when questioned about exercise, the validity of self-reported levels of physical activity was not always reliable (17). Therefore, further studies have to be done to assess the risk factors associated with a higher prevalence of obesity among female patients with DM in Yemen. More DM health education has to be provided to our patients, as the majority of them did not know the risk factors associated with the development of type 2 DM and obesity.

In this study, in the case of severe obesity (BMI ≥ 35 kg/m^2^), prevalence rate in women was about nine times higher than in males (9.7% and 1.1%, respectively), which seemed to be higher than any female-male difference in neighboring countries. Many factors have been shown to be associated with an increased risk of obesity and DM such as high intake of sugar-sweetened beverages (16, 18). In some neighboring countries, factors such as unemployment and marriage were associated with weight gain in females (19). In Saudi Arabia, working women had a lower rate of obesity than non-working ones (20); moreover, the prevalence of inactivity among people was very high (approximately 96.1%) with a significantly higher rate of inactivity among females in comparison to males. Obesity decreased with the level of education and increased with the age, especially in males (21).

DM and obesity has already become a worldwide epidemic with significant health and economic burdens (21). The best way to overcome this epidemic is screening for early detection, prevention, and early management of obesity before the development of type 2 DM, especially in younger individuals (22). Physical activity should be encouraged in our patients with DM, especially in females. The American Heart Association and the American Diabetes Association recommends carrying out at least 150 minutes of moderate-intensive aerobic activity, or at least 90 minutes of vigorous aerobic exercise per week (23)

Diet control still remains the cornerstone in the treatment of DM and most patients find this area of self-management difficult (24). It has been shown that group education for patients with type 2 DM by a diabetes specialist nurse and dietitian had better results than those receiving the usual clinic care in both weight loss and diabetes control (25). The majority of the patients claimed that they had been following what they considered as a diabetic diet. During questioning, they had many misunderstanding concerning which diet should have been prescribed. Most of the times, the diet education that they had received was prescription of a friend or a relatives who had no diabetes education or in the best situations, in a prescription from a physician. Dietary education should be emphasized in these patients. Although compliance and adherence to diet is poor among diabetics, dietary counseling has been shown to improve dietary practices in patients with type 2 DM (26). Education on the complications of obesity and DM is very important in Yemeni patients since the majority of the patients have not visited a diabetes educator or dietitian due to the low number of these professional workers. Hence, educating the patients on diet and exercise and their importance in diabetes control remains the responsibility of the physicians.

Our study had some limitations since it was a single center study with most of the patients from Sana’a and the surrounding regions. Larger studies covering the different regions of Yemen should be conducted to see if similar differences in obesity in male and female patients with DM are present. In addition, further studies have to be conducted to assess the factors causing obesity in these patients and if possible, slowing down the increasing rate of obesity and associated metabolic diseases.

In conclusion, the overweightness and obesity (high BMI) were prevalent in patients with type 2 DM in Yemen, with a higher frequency in females than in males. Moreover, the mean BMI, as a continuous variable associated with morbidity and mortality, was significantly higher in females than in males at different age groups. The finding of poor glycemic control among the majority of patients was another alarming sign of low quality diabetes care in this country. We recommend improving the standards of diabetes health care in Yemen at the primary and critical care levels in order to reduce the burden attributable to the DM in the Yemen.
